# HIF-1α Plays a Critical Role in the Gestational Sidestream Smoke-Induced Bronchopulmonary Dysplasia in Mice

**DOI:** 10.1371/journal.pone.0137757

**Published:** 2015-09-11

**Authors:** Shashi P. Singh, Hitendra S. Chand, Sravanthi Gundavarapu, Ali Imran Saeed, Raymond J. Langley, Yohannes Tesfaigzi, Neerad C. Mishra, Mohan L. Sopori

**Affiliations:** 1 Immunology Division, Lovelace Respiratory Research Institute, Albuquerque, New Mexico, United States of America; 2 COPD Program, Lovelace Respiratory Research Institute, Albuquerque, New Mexico, United States of America; 3 Pulmonary and Critical Care Medicine, University of New Mexico, Albuquerque, New Mexico, United States of America; Comprehensive Pneumology Center, GERMANY

## Abstract

**Rationale:**

Smoking during pregnancy increases the risk of bronchopulmonary dysplasia (BPD) and, in mice, gestational exposure to sidestream cigarette smoke (SS) induces BPD-like condition characterized by alveolar simplification, impaired angiogenesis, and suppressed surfactant protein production. Normal fetal development occurs in a hypoxic environment and nicotinic acetylcholine receptors (nAChRs) regulate the hypoxia-inducible factor (HIF)-1α that controls apoptosis and angiogenesis. To understand SS-induced BPD, we hypothesized that gestational SS affected alveolar development through HIF-1α.

**Methods:**

Pregnant BALB/c mice were exposed to air (control) or SS throughout the gestational period and the 7-day-old lungs of the progeny were examined.

**Results:**

Gestational SS increased apoptosis of alveolar and airway epithelial cells. This response was associated with increased alveolar volumes, higher levels of proapoptotic factors (FOXO3a, HIPK2, p53, BIM, BIK, and BAX) and the antiangiogenic factor (GAX), and lower levels of antiapoptotic factors (Akt-PI3K, NF-κB, HIF-1α, and Bcl-2) in the lung. Although gestational SS increased the cells containing the proangiogenic bombesin-like-peptide, it markedly decreased the expression of its receptor GRPR in the lung. The effects of SS on apoptosis were attenuated by the nAChR antagonist mecamylamine.

**Conclusions:**

Gestational SS-induced BPD is potentially regulated by nAChRs and associated with downregulation of HIF-1α, increased apoptosis of epithelial cells, and increased alveolar volumes. Thus, in mice, exposure to sidestream tobacco smoke during pregnancy promotes BPD-like condition that is potentially mediated through the nAChR/HIF-1α pathway.

## Introduction

Bronchopulmonary dysplasia (BPD) is the major cause of morbidity and mortality in premature babies [1, 2]. BPD is characterized by fewer and enlarged alveoli, suppressed angiogenesis, and lack or insufficient production of surfactant proteins [3]. Improved neonatal care of premature babies has led to increased numbers of babies with BPD [2, 4]. BPD-associated changes in lung function may be irreversible and linked to higher incidence of respiratory diseases later in life [4–9].

Embryonic development is highly sensitive to changes in the environment and exposure to a wide range of environmental pollutants such as cigarette smoke (CS), polycyclic aromatic hydrocarbons, and bisphenol A affect the maturation and function of the lung and contribute to the development of pulmonary diseases in children [10–14]. The risk of CS-associated pulmonary complications is the highest during fetal and early postnatal life [15, 16]. Others and we have shown that gestational exposure to CS exacerbates allergic asthma and promotes BPD in humans and animal models [12, 17–20]. In spite of the known adverse effects of gestational CS on the respiratory health of the offspring, a significant number of the prospective mothers smoke during some stage(s) of pregnancy [20, 21].

Gestational CS may also be an independent risk factor for BPD in humans [19, 22] and babies exposed *in utero* to CS, including SS, exhibit significantly lower body weight and are at higher risk of COPD/emphysema later in life [23]. The mechanism by which gestational SS induces BPD is not clearly understood. Normal angiogenesis is critical for proper alveolarization [24]. In mice, the BPD associated with gestational SS is linked to suppressed lung angiogenesis and both angiogenesis and alveolar septal formation were normalized in gestationally SS-exposed mice concomitantly treated with the nicotinic acetylcholine receptor (nAChR) antagonist mecamylamine (MM) [12].

Embryogenesis occurs in relatively hypoxic conditions [25] and the hypoxic environment is important for normal fetal development [26]. Hypoxia-controlled responses are regulated by hypoxia-induced factors (HIFs) during trophoblast formation [25] and during alveolar development and regeneration [27]. Increasing evidence suggests that CS/nicotine promotes cell growth and angiogenesis through HIF-1α [28]. HIF-1α is a transcription factor that regulates cell growth through the PI3K/Akt pathway; HIF-1α also regulates the genes that control the development of various organs including the lung [29]. Because nicotine also regulates cell growth, apoptosis, and angiogenesis through HIF-1α, we hypothesized that gestational SS impaired lung development and increased the susceptibility to BPD through HIF-1α. In this communication we present evidence that gestational SS exposure suppresses HIF-1α impacting apoptosis and lung development.

## Materials and Methods

### Animals

Pathogen-free BALB/c mice (FCR Facility, Frederick, MD) were kept in exposure chambers maintained at 26 ± 2°C and 12-hour light/dark cycle. Food and water were provided *ad libitum*. All animal protocols were approved by the Institutional Animal Care and Use Committee (IACUC).

### Antibodies and other reagents

Sources of antibodies and specific reagents used are listed in the relevant method sections.

Buffers and Precast gels for the Western blot analysis were obtained from Bio Rad Laboratories Inc. (Hercules, CA). Mecamylamine was purchased from Sigma Chemical Co. (St. Louis, MO).

### Cigarette smoke generation and exposure

Adult (3−4 month old) male and female mice were separately acclimatized to SS or filtered air (FA) for 2 weeks, and then paired for mating under the same exposure conditions. Mice were whole-body exposed to SS or FA for 6 hours/day, 7 days/week as described [12], using smoking machine (Type 1300; AMESA Electronics, Geneva, Switzerland) that generated two, 70-cm^3^ puffs/min from 2R1 cigarettes (Tobacco Health Research Institute, Lexington, KY). The dose of SS is approximately equivalent to the amount of SS a pregnant woman would receive by sitting in a smoking bar for 3 hr/day throughout the gestational period [12]. Male mice were removed after pregnancy and the pregnant mice continued to receive SS or FA until the pups were born. Mice were sacrificed on day 7 after the birth by an intraperitoneal injection of 0.2 ml Euthasol.

### Mecamylamine treatment

Where indicated, mice were exposed to mecamylamine (MM) via subcutaneously implanted model-2006 Alzet miniosmotic pumps (ALZA Corporation, CA) containing sterile saline or 2.5 mg/ml MM in sterile saline [12]. Previous studies did not show any significant effects of gestational MM on lung development [12]; therefore, the MM-alone group was not included in these experiments.

#### Preparation of lung tissues

Seven-day old mice were sacrificed, lungs were removed, and some lungs were inflated and kept in 10% formalin bath at 20 cm pressure for 24 h [12]. After washing, the lungs were embedded in paraffin and 5 μm thick tissue sections were cut and subjected to H&E staining, immunohistochemistry (IHC) and/or immunofluorescence (IF) as described previously [12]. Alveolar size (volume) was determined on H&E-stained lung sections by NanoZoomer Digital Pathology (NDP) slide scanner (Hamamatsu K. K. Photonics, HAMAMATSU City, Japan). The analysis was done blind using computer-selected random areas of the lung.

#### Immunostaining

Immunostaining for HIPK2 and bombesin-like peptides (BLP) was carried out using standard protocols. Briefly, after deparaffinization, lung sections were stained with rabbit polyclonal anti-HIPK2 antibody (cat #: ab28507, Abcam) or rabbit polyclonal anti-bombesin antibody (cat #: ab86037, Abcam). Sections were counterstained with haematoxylin.

#### Immunofluorescence staining for HIF-1α, NF-κB, activated caspase 3, cytokeratin, and cell nuclei

Lung sections were stained with anti-HIF-1α antibody (#ab16066, Abcam). HIF-1α-positive cells in FA and SS-exposed lungs were counted blind using computer selected 7000 μm^2^ areas and the NanoZoomer slide scanner. The experiment was repeated three times with different sets of animals. To score for activated NF-κB, we stained for phospho-p65-NF-κB (pRelA) using anti-pRelA antibody (# 3033, Cell Signaling Tech.). The slides were counter stained with anti-rabbit Alexa 564-conjugated seondary antibody (#A-11010, Life Technologies); nuclei were stained with DAPI (blue fluorescence). For detecting apoptotic epithelial cells, sections were costained with anti-cleaved caspase 3 (ac-Casp 3) antibody (1: 500; rabbit polyclonal, #9661, Cell Signaling Tech.) and anti-pan cytokeratin (pan-CK) antibody (1:1000; mouse monoclonal, #4545, Cell Signaling Tech.). To detect the expression of ac-Casp 3 and pan-CK, slides were counterstained with anti-rabbit Alexa 564 and anti-mouse Alexa 647-conjuagted (1:200; Life Technologies) secondary antibodies, respectively. Captured images of the stained cells were analyzed and quantified using NIH Image J software (http://rsb.info.nih.gov/nih-image/). Apoptotic cells were also detected by the TUNEL assay using *in situ* apoptosis detection kit (TACS 2 TdT-DAB; catalog # 4810-30-K) as per manufacturer’s directions (Trevigen inc., MD). TUNEL-positive cells were counted blinded.

#### Western blots

Western blot (WB) analysis of lung homogenates was carried out as described previously [30]. Briefly, tissue samples were homogenised in RIPA buffer and the protein content of the extracts was determined by the BCA Protein Assay Kit (Pierce, Rockford, IL). The homogenates were analyzed by SDS-PAGE on 10% precast polyacrylamide gels. The gels were transferred electrophoretically to nitrocellulose membranes (Bio Rad Lab, Hercules, CA) and the blots were incubated with control IgG or specific antibodies to the following proteins: acetyl p53 (Lys379, cat #: 2570S, Cell Signaling), Akt or phospho (p)-Akt (cat #: 9272S and cat #: 3787S respectively, Cell Signaling), Bcl-2 (cat #: 554087, BD Bioscience), BIK (cat #: 4592S, Cell Signaling), BIM (cat #: ab7888, Abcam), BAX (cat #: sc493, Santa Cruz Biotech), BAK (cat #: 3814S, Cell Signaling), bombesin (cat #: ab86037, Abcam), FOXO3a (cat #: ab47409, Abcam), GRPR (gastrin-releasing peptide receptor, cat #: ABR-002, Jerusalem, Israel), HIF-1α (cat #: ab1 [H1alpha67], Abcam), HIF-2α (cat #: ab20654), HIPK2 (cat #: ab28507, Abcam), PUMA (cat #: ab9643, Abcam), and NOXA (cat #: ab13687, Abcam). The mouse anti-actin antibody (Santa Cruz Biotech) was used as a control for a house-keeping protein. After incubating with secondary antibody, immunodetection was performed using Amersham ECL Western Blotting Detection Reagent (GE Healthcare Bio-Science Corp. Piscataway, NJ) and the images were captured by Fujiform LAS-4000 luminescent image analyzer (FUJIFILM Corporation, Tokyo). Densitometry was used to quantitate the expression of specific proteins in Western blots and the expression was compared to the expression of actin. Some Western blots were re-probed using alternate antibodies; thus such blots have common actin bands. For phosphorylated proteins, we calculated the densitometric ratio of the phosphorylated (p)-protein to the corresponding total protein.

### Data presentation and statistical analysis

Data were analyzed using Graph Pad Prism software 5.03 (Graphpad Software Inc., San Diego, CA). One-way ANOVA was used to compare the mean between the groups using the Tukey post-hoc test that compares all groups at 95% confidence intervals. The student’s t test was used for comparison between two groups. Results were expressed as the means (± SD). A p value of ≤0.05 was considered statistically significant.

## Results

### Gestational SS inhibits HIF-1α and the inhibition is attenuated by MM

**Fig 1:**
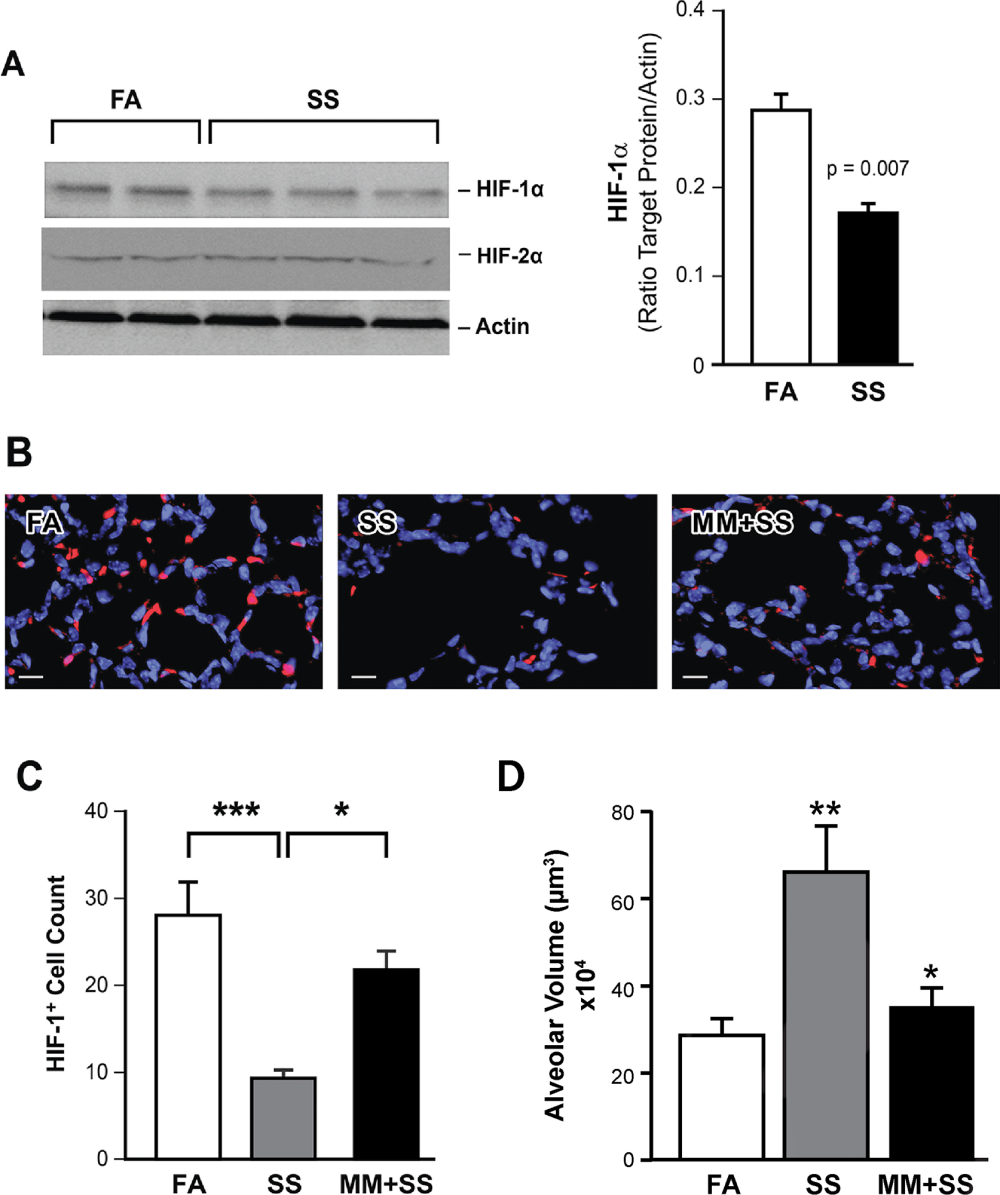
Mecamylamine attenuates the decreased expression of HIF-1α and increased alveolar volume in gestationally SS-exposed lungs. **A:** WBs of the lung homogenates (70 μg) from 7-day old mice were probed with anti-HIF-1α and anti-HIF-2α antibodies (left panel). The concentration of HIF-1α was quantitated by densitometry (right panel; n = 6). **B:** Lung sections from control (FA), SS, and MM+SS were stained with anti-HIF-1α antibody followed by anti-rabbit Cy3 conjugated secondary antibody. Slides were counterstained with DAPI for nuclei. Images are representative of n = 6/group. HIF-1α (red) and nuclei (blue); scale bar = 10 um. **C:** Graphical representation of HIF-1α^+^ cells. Cells were counted blind over the randomly computer selected 7000 μm^2^ areas by NDP. The experiment was repeated three times with different sets of animals. *, p ≤ 0.05; ***, p ≤ 0.001. **D:** Graphical representation of the differences in alveolar volumes between FA, SS, and MM+SS in 7-day old lungs. Data are presented as mean ± SD (*n* = 5). MM+SS vs FA = ns; MM+SS vs SS, p *≤* 0.05; SS vs FA, p ≤ 0.01.

Hypoxia is essential for the early fetal development and adaptation to hypoxia is primarily regulated by HIFs [25,26] and, in multiple cell types, the expression of HIF-1α is regulated by nAChRs [28]. We determined whether gestational SS affected HIF-1α and HIF-2α in the 7-day old lung and, if so, whether the effects were regulated by nAChRs. Western blot analysis of the 7-day old lung extracts indicated that, compared to control lungs, HIF-1α but not HIF-2α protein is significantly reduced in the lung extracts from SS-exposed animals (Fig 1A). Moreover, immunofluorescence studies showed that the lung sections from gestationally SS-exposed animals had reduced HIF-1α-positive staining (Fig 1B) and the number of HIF-1α-positive cells (Fig 1C). However, the SS-induced reduction in HIF-1α-positive cells was significantly moderated in the lungs of animals whose mothers were treated simultaneously with SS and the nAChR antagonist MM (Fig 1B and 1C). In addition, as determined by the changes in alveolar volumes, MM treatment also attenuated the SS-induced alveolar simplification in the 7-day old lung (Fig 1D). These results suggest a potential role of nAChRs in SS-induced changes in HIF-1α and normal alveolar development.

### Gestational SS induces apoptosis in epithelial cells lining the airways and alveoli

**Fig 2:**
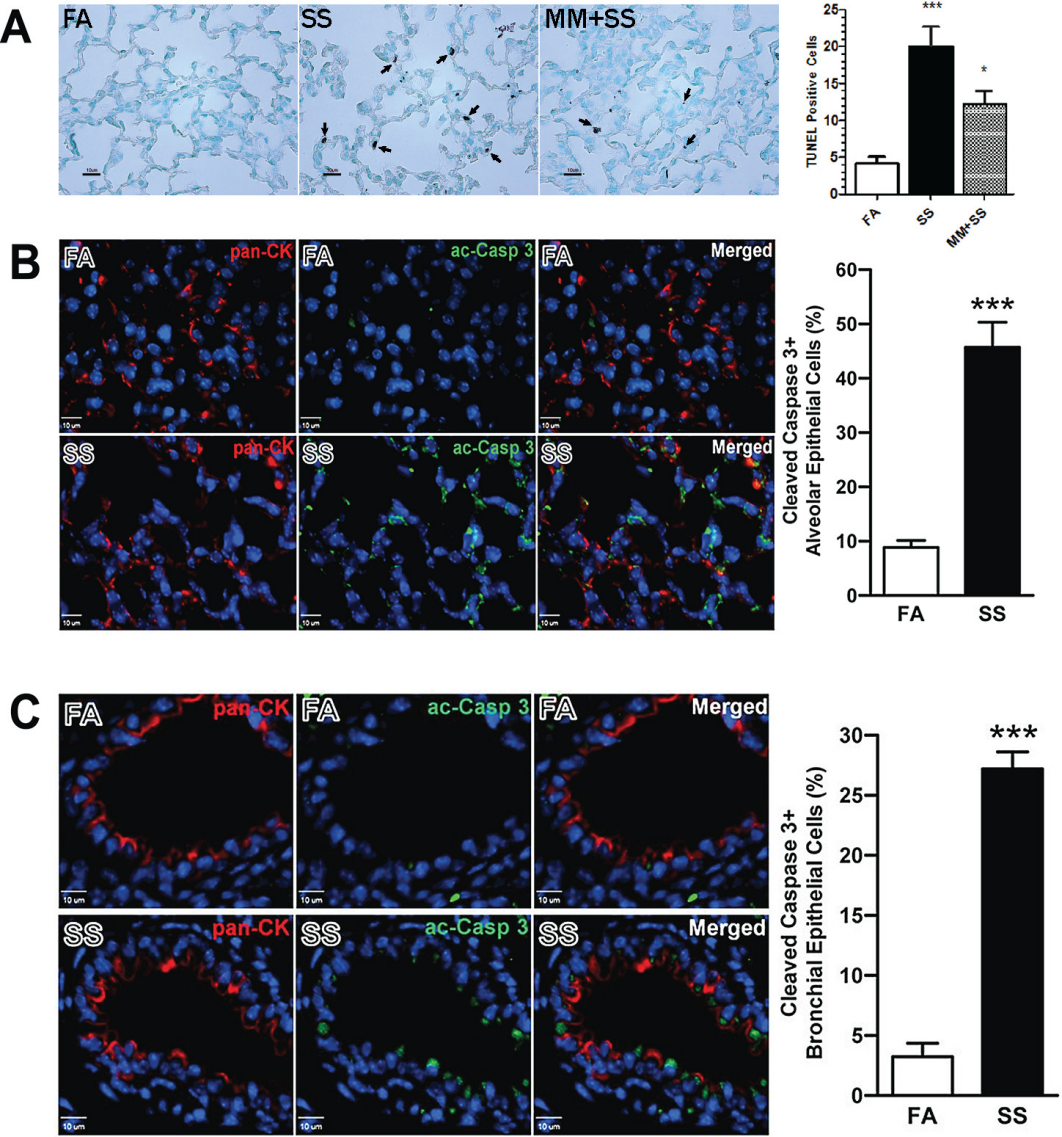
Gestational SS causes apoptosis in epithelial cells of airways and alveoli. Seven-day old lung sections from FA, SS, and MM+SS mice were examined for apoptosis by TUNEL staining. Representative micrographs showing: **A:** TUNEL-positive cells in the alveolar region. Right panel: bar graph of TUNEL positive cells (n = 6/group). **B**: Alveolar region of 7-day old lungs from FA and SS mice were analyzed for ac-Casp 3 (green) and pan-CK (red) and graphed (left panel). **C:** pan-CK and ac-Casp 3staining of the airways in FA and SS animals. In **B** and **C**, lung sections were counterstained with DAPI (blue). n = 3/group; *, p ≤ 0.05, ***, p ≤ 0.001.

In addition to defective alveolar septation, gestational SS decreases the production of surfactant producing cells, suggesting that the airway epithelial cells are a potential target of gestational SS [12]. Therefore, we ascertained whether gestational SS affected the survival of the airway and alveolar cells. Lung sections from 7-day old FA- and SS-exposed animals were examined for apoptotic cells by TUNEL staining. While the lung sections from control animals have very few TUNEL-positive cells, the alveolar region from SS-exposed lungs have significantly more TUNEL-positive cells and this number is significantly decreased in the 7-day old lungs from animals where dams were treated with SS + MM (Fig 2A). Thus, gestational SS promotes apoptosis in the lung that is potentially mediated by nAChRs as evidenced by decreased TUNEL expression in MM-treated animals. To ascertain the location and the type of cells undergoing apoptosis in the SS-exposed lung, we used lung sections covering the alveolar region (Fig 2B) and small airways (Fig 2C) and examined the sections by IF staining for the presence of the epithelial cell marker pan-CK (red) and the apoptotic marker cleaved (activated) caspase 3 (ac-Casp 3: green). It is clear that the lung airways and alveoli from gestationally SS-exposed animals have significantly more apoptotic (ac-Casp 3-positive) cells which co-express the epithelial cell marker (pan-CK). Thus gestational SS promotes apoptosis of epithelial cells in the airways and alveoli and this apoptotic response is potentially regulated by nAChR.

### Gestational SS downregulates antiapoptotic factors

**Fig 3:**
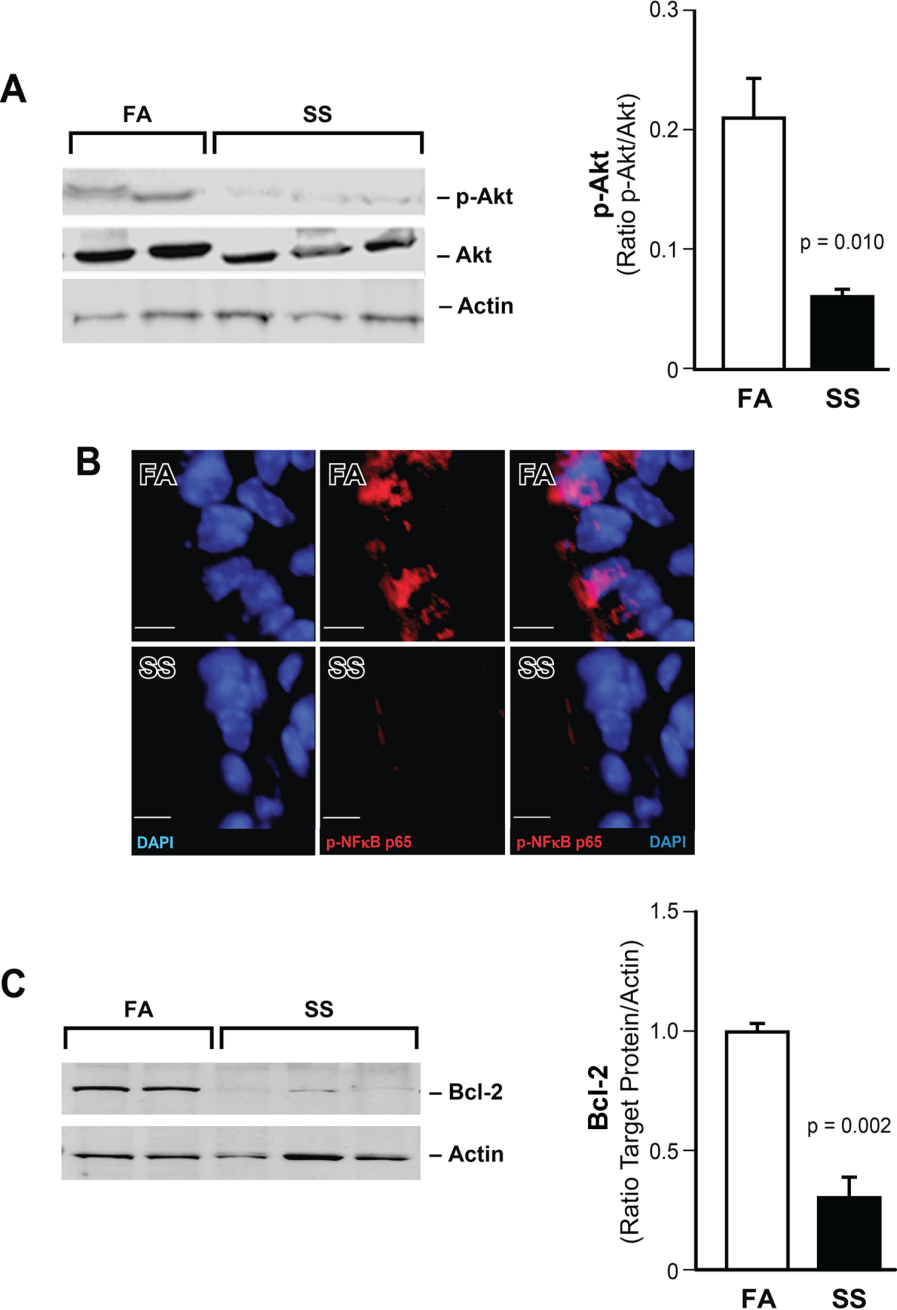
Gestational SS downregulates antiapoptotic factors in the lung. **A**: Representative WB of lung homogenates (70 μg) from FA and SS-exposed animals. The blots were developed for Akt and pAkt as described in the methods section. Right panel: densitometry of the blot (FA, n = 4; SS, n = 6). **B:** A representative micrograph of bronchial epithelium showing DAPI (blue), pRel A (red), and DAPI/pRel merged images. Scale bar = 5 μm; n = 4/group. **C:** A representative WB probed with anti-Bcl-2 antibody. Right panel is densitometry of the blots (FA, n = 4; SS, n = 6).

HIF-1α promotes cell growth and inhibits apoptosis [31]. To ascertain whether gestational SS-induced reduction in HIF-1α affected apoptotic processes in the lung, we examined pro- and antiapoptotic factors in FA and SS-exposed 7-day old lungs. HIF-1α is activated by the antiapoptotic kinase, phosphorylated-Akt (pAkt) [32,33] through phosphorylation of RelA (p65-NF-κB) [30]. We determined the expression of pAkt by Western blot analysis and the presence of pRelA by IF staining in FA and gestationally SS-exposed lungs. The results indicate that compared to total Akt, pAkt is markedly reduced in the gestationally SS-exposed lung (Fig 3A). Similarly the density of immunoreactive pRelA is reduced in SS-exposed lungs (Fig 3B). Double labeling for pRelA (red) and cell nuclei (DAPI, blue) indicated that pRelA is associated with DAPI in FA but not in SS-exposed lungs (Fig 3B). In addition, Western blot analysis indicates that the antiapoptotic factor Bcl-2 is significantly decreased in SS-exposed samples (Fig 3C). Together these results suggest that along with HIF-1α, the antiapoptotic factors pAkt, pRelA, and Bcl-2 are decreased in gestationally SS-exposed lungs.

### Gestational SS upregulates proapoptotic factors

**Fig 4:**
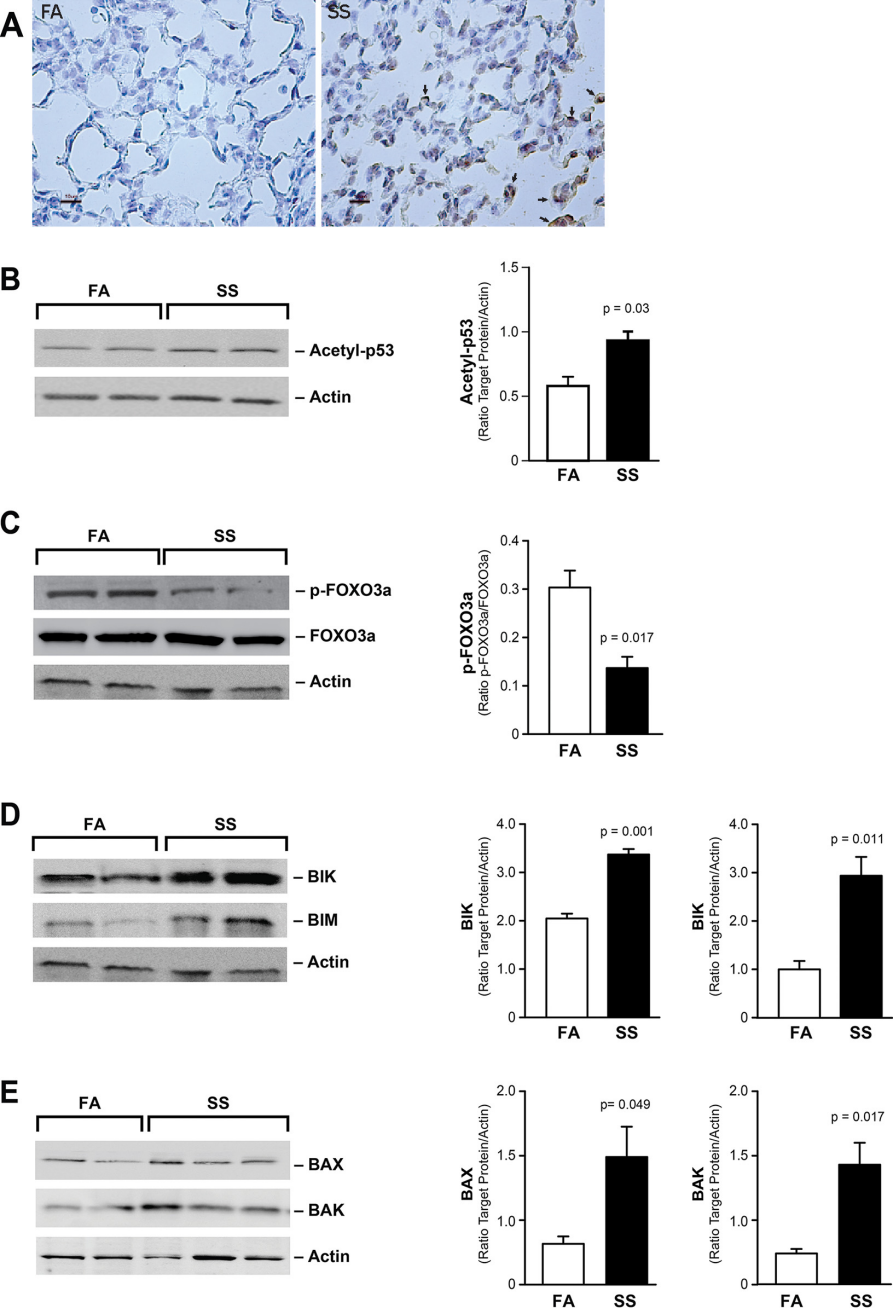
Gestational SS upregulates proapoptotic factors. **A:** Representative IHC staining for HIPK2 in FA and SS lungs. **B:** Representative WB of lung homogenates (70 μg) using antibody specific for acetylated p53 (Lys379); densitometry of the blot (**right** panel). **C**: WB probed with antibodies to p-FOXO3a and FOXO3a> Densitometry of p-FOXO3a (right panel; n = 4/group). **D:** WB probed with anti-BIK and anti-BIM antibodies. Densitometry of BIK and BIM blots (right panel). **E:** WB probed with anti-BAX and anti-BAK antibodies; densitometry of BAX and BAK (right panel). Experiments in A, B, C, and D had n = 4 for FA and SS; E had FA, n = 4 and SS n = 6.

To ascertain whether the decrease in HIF-1α increases the levels of proapoptotic factors in SS-exposed lungs, we examined the expression of proapoptotic factors that are known to be regulated by HIF-1α. As detected by IHC staining, compared to FA, the expression of proapoptotic homeodomain-interacting protein kinase-2 (HIPK2) is strongly upregulated in gestationally SS-exposed lungs (Fig 4A). HIPK2 induces apoptosis through activation of p53 [34] that, in mice, involves acetylation of p53 at Lys379 [35]. WB analysis showed that the concentration of acetylated Lys379 containing p53 is significantly higher in the lung extracts from gestationally SS-exposed animals (Fig 4B). Thus the proapoptotic factors HIPK2 and activated p53 are increased in gestationally SS-exposed lungs.

HIF-1α also regulates the activity of other proapoptotic factors, including the Forkhead transcription factor (FOXO3a) and BH3-only proapoptotic factors. The active form of FOXO3a is unphosphorylated and the WB analysis shows that the phosphorylated form of FOXO3a is higher in FA samples (Fig 4C). FOXO3a and p53 primarily control apoptosis by activating BIM [36] and BIK [37]. We determined the levels of several proapoptotic factors that mediate the “intrinsic” apoptotic pathway including the BH3-only members PUMA, NOXA, BIK, and BIM, as well as the BH4 members BAX and BAK. WB blot analysis indicates that the concentrations of BIK and BIM are higher in SS than FA-exposed lung (Fig 4D); however, the levels of PUMA and NOXA are not affected by gestational exposure (not shown). BH3-only proteins mediate apoptosis through oligomerization with BAX and BAK [37], and the lungs from gestationally SS-exposed animals have higher levels of BAK and BAX (particularly BAK) (Fig 4E). These results suggest that gestational SS upregulates several pro-apoptotic factors in the lung.

### Gestational SS stimulates antiangiogenic factors

**Fig 5:**
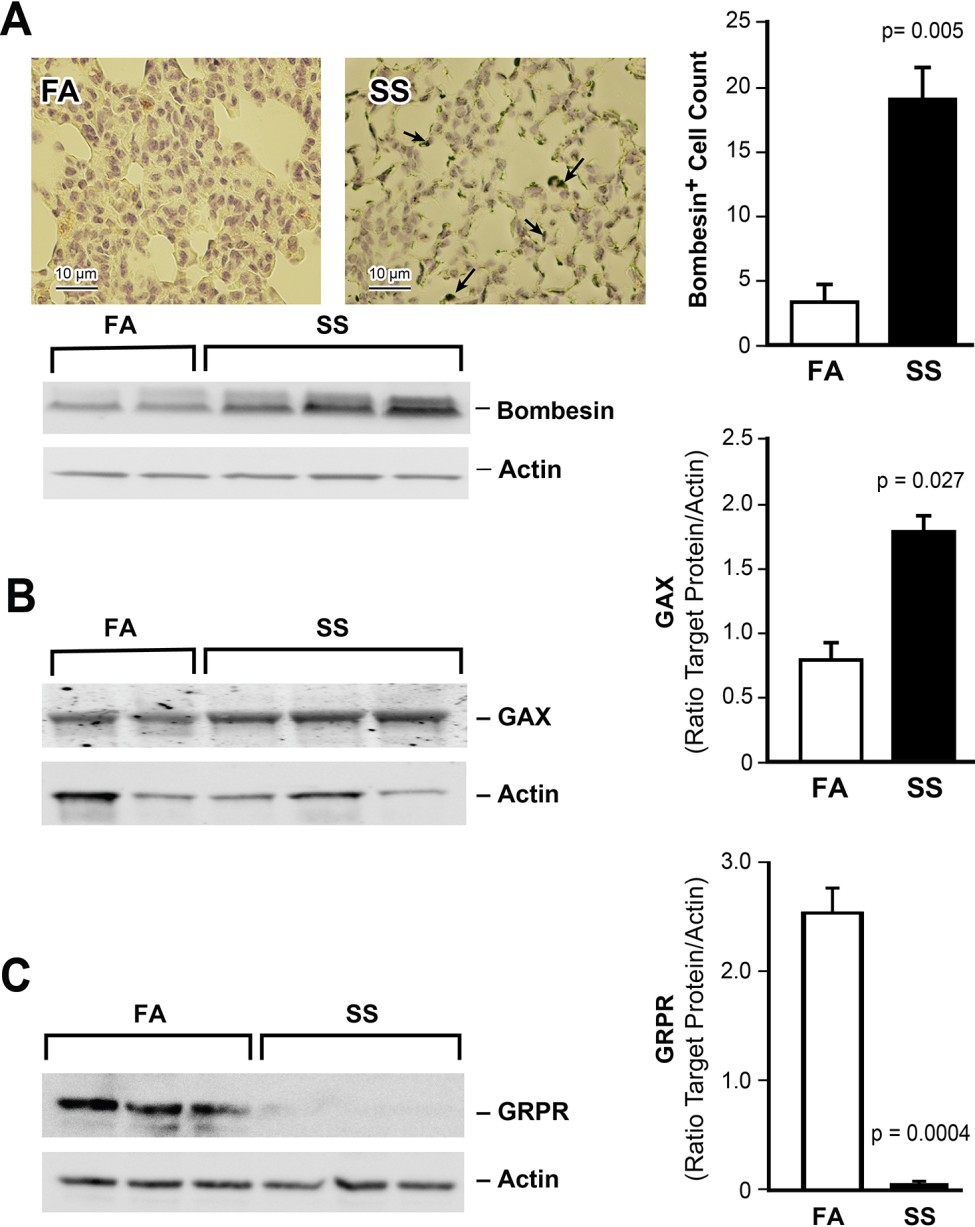
Gestational SS stimulates bombesin-like peptide (BLP) and Gax in the lung. **A:** IHC staining of lung sections using anti- BLP antibody (left), quantitation (right), and WB of lung homogenate (70 ug) probed with anti-BLP antibody (below). Bombesin-positive cells were counted in computer-selected random lung sections of 6000 um area. The images are representative of n = 3/group. **B:** WB (70 ug lung homogenate) probed with anti-GAX antibody; densitometry of GAX (right panel). **C:** WB (70 ug lung homogenate) probed with anti-GRPR antibody; densitometry of GRPR (right panel). For panels **B** and **C:** FA, n = 4; SS, n = 6.

Angiogenesis is critical in early lung development and gestational SS suppresses the proangiogenic factors VEGF and VEGFR2 in the lung [12]. In the developing lung, angiogenesis is positively regulated by HIF-1α [29] and negatively by the transcription factor GAX [38] and bombesin-like neuroendocrine peptides (BLP) [39, 40]. Therefore, we examined the expression of BLP and GAX in FA and gestationally SS-exposed lungs. IHC staining did not detect significant BLP-positive cells in control; however, a large number of BLP immunoreactive cells are seen in SS-exposed lungs (Fig 5A, upper left and right panels). Moreover, WB analysis shows higher concentrations of bombesin protein in the lungs of gestationally SS-exposed animals (Fig 5A, lower panel). Western blot analysis also shows higher levels of GAX protein in SS-exposed lungs (Fig 5B). Thus, gestational SS increases the level of BLP and GAX. Paradoxically, in adult animals, BLP is linked to increased angiogenesis [41] and the proangiogenic activity is primarily mediated through the gastrin-releasing peptide receptors (GRPR) [42]. As seen in Fig 5C, GRPR is markedly downregulated in SS-exposed lungs suggesting that, although gestational SS increases BLP, it decreases the expression of its main receptor GRPR in the lung.

## Discussion

The “fetal origins” hypothesis proposes that *in utero* exposures to toxic xenobiotics at critical periods during organogenesis cause long-term physiological and/or metabolic changes in the fetus, contributing to disease development at later age [43]. Indeed, gestational but not postnatal exposure to low levels of environmental toxins, such as bisphenol A [13] and polycyclic aromatic hydrocarbons [10] affect lung development and increase susceptibility to lung diseases. There is now considerable evidence that in humans and animals, *in utero* exposure to cigarette smoke including SS promotes respiratory diseases postnatally [12, 22, 23, 30] and, in our experiments, the gestational SS exposure of mice leads to airway hyperreactivity and BPD. The amount of SS that causes these diseases in mice translates to the human equivalent of smoking approximately half a cigarette/day[12].

Previous studies from our laboratory indicated that gestational exposure to SS cause a BPD-like condition in mice characterized by a significant increase in alveolar size and decreased surfactant production that is essentially blocked by the nAChR antagonist MM [12]. The role of nAChRs on cell growth has been extensively studied in tumor cells, where the activation of nAChRs decreases apoptosis and increases angiogenesis and tumor growth [28]. Interestingly, lungs from gestationally SS-exposed animals have a significantly higher number of TUNEL-positive and activated caspase 3-positive cells in the airways and in the alveolar region. Moreover, the activated caspase 3-positive cells are also positive for the epithelial cell marker cytokeratin, indicating that the gestational exposure to SS induces apoptosis of epithelial cells in the airways and alveoli. Normally, the activation of nAChRs by CS/nicotine promotes cell growth and differentiation by inhibition of apoptosis [28, 44]; paradoxically, however, gestational exposure to SS appears to increase TUNEL-positive cells in the postnatal lung and the increase is reduced in MM-treated animals suggesting the possibility that the gestational SS-induces proapoptotic response that is potentially mediated through nAChRs. However, this is a preliminary inference that requires more definitive experiments involving different concentrations of MM and larger number of animals for confirmation.

Hypoxia is one of the fundamental biological phenomena that controls cell growth and differentiation, and HIF-1α functions as a master transcription factor regulating the hypoxia responsive genes that are important in the development of multiple organs [45, 46], including the lung [29, 47]. Normal fetus develops under relatively hypoxic conditions and HIF-1α plays a critical role in the intrauterine alveolar differentiation and surfactant production [48, 49]. In tumor cells, the antiapoptotic and proangiogenic effects of nicotine have been attributed to its ability to stimulate HIF-1α through nAChRs [28]. Therefore, if SS were to activate nAChRs, it would be expected to stimulate proangiogenic and antiapoptotic responses. Surprisingly; however, gestational SS promoted apoptosis and inhibited angiogenesis in the developing lung. Therefore, to understand the mechanism of this paradoxical effect, we ascertained the level of HIF-1α in the 7-day old lung from FA and SS-exposed animals. We observed that compared to FA, the expression of HIF-1α is significantly lower in gestationally SS-exposed lungs, and the effect is attenuated by concomitant treatment with mecamylamine. We have previously shown that gestational SS is associated with defective alveolar septation and increased mean linear intercept in alveoli that is attenuated in animals exposed to SS + MM [12]. In these studies, the effect of MM on the alveolar architecture in SS-exposed animals was confirmed by assessing alveolar volumes of 7-day old lungs, which suggested that MM blocks SS-induced changes in HIF-1α and alveolar volumes. Thus, nAChRs are potentially involved in lung development through HIF-1α; however, contrary to the observations in tumors, gestational exposure to cigarette smoke reduces HIF-1α in the neonatal lung and may contribute to the increased apoptosis and decreased lung angiogenesis in these animals. Nicotinic acetylcholine receptors are complex structures that are stabilized positively or negatively by a number of different proteins [50] and, it is likely, that constant presence of nicotine through SS exposure desensitizes nAChRs leading to the effects that are opposite to those seen after normal “activation” of nAChRs. Indeed, in vivo, MM was shown to upregulate the surface expression of nAChRs and may increase their stability[51]. While we have no direct evidence to suggest that chronic gestational SS desensitizes nAChRs, this is a likely explanation for the paradoxical proapoptotic/antiangiogenic effects of SS in the developing lung.

Hypoxia improves cell survival through induction of HIFs and HIF-1α promotes cell survival by upregulating the antiapoptotic factors pAkt and activating NF-κB [52]. Indeed, HIF-1α increases pAkt [32, 33] and protects alveoli from injury [53]. Conversely, inhibition of HIF-1α decreases pAkt [32] and increases the level of proapoptotic factor FOXO3a [54]. Our results show that gestational SS downregulates pAkt and pRelA and this decrease is associated with the increased cell apoptosis in gestationally SS-exposed lungs. An established mechanism by which HIF-1α suppresses apoptosis is by degradation of the highly conserved serine/threonine kinase HIPK2 [34] and, in our studies, the level of HIPK2 was significantly increased in gestationally SS-exposed lungs. HIPK2 stimulates apoptosis through activation of the tumor suppressor protein p53 via its acetylation at Lys379 in mice [55] and, indeed, acetylation of p53 at Lys379 is increased in gestationally SS-exposed lungs. Although p53 and FOXO3a are affected by HIF-1α and use similar BH3-only factors to cause apoptosis, they are not necessarily interdependent [56]. Of the proapoptotic factors tested, BIK, and BIM (BH3-only) and BAX and BAK (BH4) were significantly upregulated, but PUMA and NOXA were not significantly affected by gestation SS. BIK and BIM are known to cooperate in the induction of cell apoptosis [57]. BIK closely associates with p53 and BIM is primarily regulated by FOXO3 [58], and both BIK and p53 stimulate the recruitment of BAX and BAK to trigger apoptosis [37]. It should be emphasized that this balance between pro- and anti-apoptotic factors in FA and SS-exposed lungs has been determined only in the 7-day postnatal lungs and whether this condition persistent beyond this period is not known at present.

BPD is associated with impaired angiogenesis [24] and we have shown that gestational SS downregulates VEGF and VEGFR2 in the lung [12]. A recent study concluded that the enactment of tobacco-free laws have led to a significant reduction in low birthweight babies [59] and a large epidemiological study showed that the vascular changes caused by exposure of children to parental SS are permanent and seen even at 25 years after the exposure [60]. The mechanism by which gestational SS regulates angiogenesis in the developing lung is not clear; however, angiogenesis is critical for normal alveolarization [61]. Our results indicate that gestational SS downregulates the proangiogenic factors HIF-1α and NF-κB but upregulates the expression of antiangiogenic transcription factor GAX. GAX is known to suppress angiogenesis through inhibition of NF-κB activation [38]. At present we do not have direct evidence to show that GAX affects angiogenesis through changes in NF-κB; nonetheless, NF-κB is upstream of HIF-α and responds to changes in Akt/ERK1/2 activity [52, 62]. Another factor that impacts lung angiogenesis is BLP. BLP inhibits NF-κB activation and angiogenesis in the developing lung [38, 40]. Normally, BLP promotes angiogenesis by binding to GRPR [63]; however, our results suggest that the expression of GRPR is markedly reduced in the lungs of gestationally SS-exposed animals. Thus the lack of appropriate receptors may hinder the proangiogenic effects of BLP. It is conceivable that the high level of BLP is a compensatory mechanism to improve angiogenesis and alveolarization in gestationally SS-exposed lungs. Interestingly, BLP-containing neuroendocrine cell hyperplasia in infancy is also associated with permanent changes in expiratory airflow [64] and is more prevalent in children who survive BPD [8, 9]. Together, these studies suggest that BPD-like changes in the lung induced by gestational exposure to SS are related to the changes in apoptotic pathways controlled by HIF-1α and potentially regulated by nAChRs.

**Fig 6:**
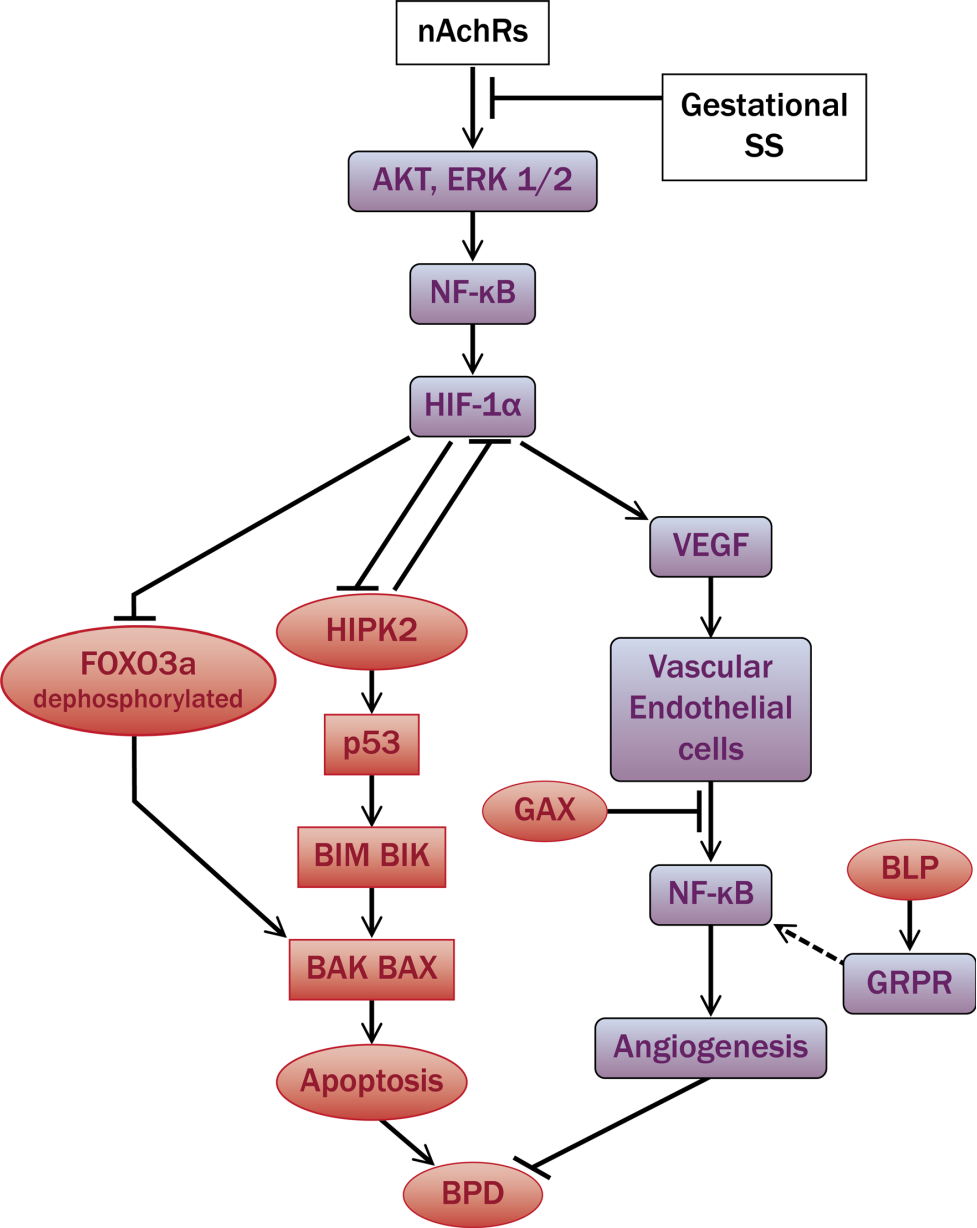
Schematic diagram of the pathway that potentially links gestational SS to HIF-1α and BPD. The pathway shows the potential effects of “normal” activation of nAChRs by nAChR ligands that increase the level of antiapoptotic factors AKT, ERK1/2 that in turn promote accumulation of HIF-1α through activation of NF-κB. HIF-1α inhibits the proapoptotic factors HIPK2 and FOXO3a leading to downregulation of BAX and inhibition of apoptosis. HIF-1α also promotes angiogenesis through increased VEGF and decreased GAX production. NF-κB is critical for normal alveolar septation and inhibits BLP. The proangiogenic effects of BLP require GRPR that are downregulated in gestationally SS-exposed lungs. In gestationally SS-exposed animals, lungs exhibit increased apoptosis and decreased angiogenesis, and seen as upregulated (red) expression of proapoptotic and antiangiogenic factors and downregulated (blue) expression of anti-apoptotic and proangiogenic factors, leading to a BPD-like condition. The interaction between GRPR and NF-κB is hypothesized and connected by a dashed line.

There is evidence that inflammation in the early postnatal lung promotes BPD [65, 66]; however, in gestationally SS-exposed 7-day mouse lung, there is no indication of infiltrating leukocytes, suggesting the lack of significant inflammatory response in this model of BPD. While it is possible that the BPD-like phenotype exhibited by gestationally SS-exposed mice does not totally replicate human BPD, anti-inflammatory drugs have not proven beneficial in preventing BPD in humans and experimental animals [67–69]. Even CCSP (rhCC10) that reduces lung inflammation [70] does not decrease the risk for BPD in preterm infants [71]. Moreover, a recent study failed to observe a significant correlation between proinflammatory cytokines in the tracheal aspirates and development of BPD in preterm infants [72]. On the other hand, while there was no difference in cell count and IL-8 content in tracheobronchial lavages from preterm babies immediately after the birth, subsequently BPD was associated with increased cell infiltration and IL-8 in the their lavages [73]. In rabbit models of BPD, some studies show a relationship between lung inflammation and BPD, while others fail to see the relationship [74]. Therefore the role of inflammation in BPD is not totally clear and commonly used interventions that improve survival of preterm infants (e.g., hyperoxia) may promote lung inflammation and facilitate the development of BPD. In gestationally SS-exposed mice, changes in alveolarization and angiogenesis are not associated with detectable inflammation but, as we have shown previously, these mice are highly susceptible to allergen-induced lung inflammation [30]. It is likely that the BPD phenotype arises through the effects of gestational SS on lung development and these developmental defects are casually related to the HIF-1α pathway, which might be regulated by nAChRs. A schematic diagram showing the potential relationship of HIF-1α to the apoptotic and angiogenic pathways in gestationally SS-exposed lungs is presented in Fig 6.
